# Silencing MicroRNA-134 Alleviates Hippocampal Damage and Occurrence of Spontaneous Seizures After Intraventricular Kainic Acid-Induced Status Epilepticus in Rats

**DOI:** 10.3389/fncel.2019.00145

**Published:** 2019-04-12

**Authors:** Xiaoying Gao, Mian Guo, Dawei Meng, Feixiang Sun, Lianyue Guan, Ying Cui, Yan Zhao, Xichun Wang, Xin Gu, Jiahang Sun, Sihua Qi

**Affiliations:** ^1^Department of Anesthesiology, The Fourth Affiliated Hospital of Harbin Medical University, Harbin, China; ^2^Department of Neurosurgery, The Second Affiliated Hospital of Harbin Medical University, Harbin, China; ^3^Department of Neurosurgery, Aviation General Hospital of China Medical University, Beijing, China; ^4^Department of Hepatobilary-Pancreatic Surgery, China-Japan Union Hospital of Jilin University, Changchun, China; ^5^Department of Radiotherapy, Cancer Hospital of Harbin Medical University, Harbin, China; ^6^Department of Neurosurgery, Heilongjiang Provincial Hospital, Harbin, China; ^7^Department of Head and Neck Surgery, Cancer Hospital of Harbin Medical University, Harbin, China

**Keywords:** microRNA-134, hippocampal neurons, oxidative stress, epilepsy, CREB

## Abstract

Epilepsy is a disorder of abnormal brain activity typified by spontaneous and recurrent seizures. MicroRNAs (miRNAs) are short non-coding RNAs, critical for the post-transcriptional regulation of gene expression. MiRNA dysregulation has previously been implicated in the induction of epilepsy. In this study, we examined the effect of silencing miR-134 against status epilepticus (SE). Our results showed that level of miR-134 was significantly up-regulated in rat brain after Kainic acid (KA)-induced SE. TUNEL staining showed that silencing miR-134 alleviated seizure-induced neuronal apoptosis in the CA3 subfield of the hippocampus. Western blot showed that a miR-134 antagonist suppressed lesion-induced endoplasmic reticulum (ER) stress and apoptosis related expression of CHOP, Bim and Cytochrome C, while facilitated the expression of CREB at 24 h post KA-induced lesion in the hippocampus. Consistently, silencing miR-134 significantly diminished loss of CA3 pyramidal neurons using Nissl staining as well as reducing aberrant mossy fiber sprouting (MFS) in a rat epileptic model. In addition, the results of EEG and behavior analyses showed seizures were alleviated by miR-134 antagonist in our experimental models. These results suggest that silencing miR-134 modulates the epileptic phenotype by upregulating its target gene, CREB. This in turn attenuates oxidative and ER stress, inhibits apoptosis, and decreases MFS long term. This indicates that silencing miR-134 might be a promising intervention for the treatment of epilepsy.

## Introduction

Epilepsy is a common, chronic neurologic disorder characterized by recurrent spontaneous seizure, which affects an estimated 50 million people globally. The already existing anti-epileptogenic therapeutics have exhibited limited clinical efficacy, with a significant fraction of epilepsy patients remaining refractory to treatment (Jimenez-Mateos et al., 2012). Although a large number of studies using clinical neuroimaging in conjunction with experiments in model systems have indicated that seizures may cause neuronal death under certain circumstances (Ben-Ari, 1985; Henshall and Simon, 2005), our current understanding of the mechanistic processes underlying seizure-related cell death remains poorly understood.

The excitotoxicity is always thought to be one of the leading causes of cell death in animal models of epilepsy and humans (Olney et al., 1986). Recent studies have shown that oxidative stress, defined as a disruption of pro-oxidant/anti-oxidant balance, is potentially toxic for neuronal cells and increases the production of reactive oxygen species (ROS) and redox (Uttara et al., 2009). The aberrant ROS levels and redox balance can result in endoplasmic reticulum (ER) dysfunction, ER stress, and the unfolded protein response, which play a fundamental role in the pathogenesis of epilepsy. Thus, antioxidants may be new therapeutic options in seizure related diseases. Previous studies have attempted to develop effective therapeutics by targeting and modulating apoptotic pathways, particularly in neural model systems. CHOP, known as the marker of the ER stress, is responsible for most ER stress-related-apoptosis. cAMP-responsive element-binding protein (CREB), a ubiquitously expressed transcription factor, controls proliferation and differentiation during neural development, and also functions as a key regulator of neuronal plasticity, learning, and memory in the adult brain. CREB exerts profound neuro-protective actions against various insults, including ischemia, traumatic injury, and neuro-degeneration.

MicroRNAs (miRNAs) are an evolutionarily conserved class of short non-coding RNAs that post-transcriptionally regulate gene expression, which play critical roles in normal development, the establishment of cell fate, and myriad cellular processes in all organs, particularly the brain (Ouyang et al., 2013). Loss or aberrant function of miRNAs has been linked to a diverse array of brain disorders, such as epilepsy (Jimenez-Mateos et al., 2015). A study by Guo J. et al. (2014), revealed a possible pathogenetic role of phosphorylation of CREB (p-CREB)/miRNA-132 (miR-132) signal pathway in temporal lobe epilepsy (TLE) by modulating the dendritic plasticity. Huang et al. (2014), pointed out that microRNA-132 silencing decreases the spontaneous recurrent seizures. Aronica et al. (2010), also demonstrated that expression of miR-146a was involved in the modulation of the astroglial inflammatory response occurring in TLE. In addition, Cai et al. (2016), demonstrated that miR-155 antagonist alleviated of epilepsy by induction of the expression of brain-derived neurotrophic factor (BDNF). Previous studies have revealed the neuroprotective effect of a miR-134 antagomir, which can reduce ischemic injury (Huang et al., 2015) and cause prolonged seizure suppression. Moreover, downregulation of miR-134 attenuates hydrogen peroxide (H_2_O_2_)-induced retinal ganglion cell (RGC) apoptosis.

In this study, we examined whether miR-134 was involved in protecting neurons from oxidative stress-induced damage and thereby improving the long-term symptoms and progression of occurrence of spontaneous seizures.

## Materials and Methods

### Ethics Statement

All animal work was performed in accordance with the Chinese Animal Welfare Act and all experiments involving animals were approved by the Medical Ethics Committee of the Second Clinical College of Harbin Medical University (Approval ID: 201001).

### Animals

Animal experiments performed in compliance with the National Institutes of Health guidelines for the use of laboratory animals. Adult male Sprague-Dawley rats weighing 210–250 g, were obtained from the Harbin Medical University 2nd Affiliated Hospital Laboratories, China, and housed with a 12-h light/dark cycle. Rats were given food and water *ad libitum* for at least 1 week before all experiment. All experiments were performed in the morning.

### Seizure Models

Rats were anesthetized with isoflurane (5% induction, 1–2% maintenance), then immobilized in a stereotaxic apparatus (WPI Stoelting, United States) with a plane of incision bar set at 3.3 ± 0.33 mm below the interaural line. After making a midline incision, the skull was exposed and one burr hole was drilled according to the coordinates: 3.7 mm posterior to the bregma, 4.1 mm lateral to the midline, and 3.5 mm under the dura. Each rat was injected with 1.0 μL of KA (0.5 μg in 1.0 μL Saline) (Sigma-Aldrich) in the right lateral ventricle at the speed of 0.2 μL/min. The epileptic rats were further used for experiments (Xie et al., 2011). Duration and severity of seizure activity were documented. The behaviors of the seizure models were scored as Racine’s Scale Evaluation (Racine et al., 1972). Only rats exhibiting at least 1 h of continuous seizures graded at stage 4/5 were included in this study. After application of KA for 60 min, all rats received lorazepam (Ativan, 10 mg/kg, i.p.) to terminate seizures. All rats were euthanized at 24 h or 2 months following anesthesia with isoflurane, and brain tissue was stored for subsequent analysis.

### Intracerebroventricular Injections

Intracerebroventricular injections was performed as previously described (Antagomirs Targeting MicroRNA-134 Increase Limk1 Levels After Experimental Seizures *in Vitro* and *in Vivo*). Briefly, after controlled by diazepam administration, the SE rats were immediately anesthetized with sodium pentobarbital (30 mg/kg) and placed in a stereotaxic frame. The rats were inserted with a 23-gauged stainless-steel guide cannula into the bilateral ventricle through a hole drilled through the skull 4.4 mm below the top of the skull, 1.5 mm lateral and 0.8 mm posterior to the bregma. Rats received 2 μL infusion of either vehicle (normal saline) or antagomirs targeting miR-134 (Ant-134: 5′-CC CCUCUGGUCAACCAGUCACA-Chol-3′) (full length nucleotide 2′-methoxy modification, GenePharma, Shanghai, China) in artificial cerebrospinal fluid at a speed of 0.2 μL/min. The cannula was remained in the brain for additional 10 min. Rats were sacrificed at the indicated time for further analyses.

### Animal Grouping

A total of 144 rats were randomly divided into four groups (*n* = 36 per group). For the SE+ant-134 group (SE+ant-134), antagomir. Rats in the SE+vehicle group (SE+vehicle) were stereotaxically injected with KA plus the equal volume of normal saline. Rats in the control group (control) were stereotaxically injected with the equal volume of normal saline. Six rats were used for PCR and western blot (24 h) in each group, 5 for TUNEL assays (24 h) in each group, 6 for PCR (7 days) in SE and SE+ant-134 groups, 6 for PCR (2 m) in SE and SE+ant-134 groups, 4 for Nissl staining (2 m) in all groups, four for Timm staining (2 m) in four groups, four for EEG and behavioral study (2 m) in SE and SE+ant-134 groups. In the control group, 19 rats were sacrificed (11 at 24 h and 8 in 2 m), and the remaining 17 rats were killed. The SE group, 35 rats were sacrificed (11 at 24 h, 6 on 7 days and 18 in 2 m), and 1 died. In the SE+ant-134 group, 35 rats were sacrificed at the end of the experiment (11 at 24 h, 6 on 7 days and 18 in 2 m) and 1 died. In the SE+vehicle group, 35 rats were sacrificed (11 at 24 h, 6 on 7 days and 18 in 2 m) and 1 died. All rats were euthanized at 24 h or 2 months following treatment with isoflurane, and brain tissue was stored for subsequent analysis.

### Rat Brain Tissue Processing

Rats were sacrificed by decapitation after anesthetized with chloral hydrate (350 mg/kg, i.p.). The hippocampus was dissected, and then stored in liquid nitrogen for Western blot assay and Real-time quantitative polymerase chain reaction (RT-qPCR). The remaining rats in each group were anesthetized and perfused with normal saline followed by post-fixation in 4% paraformaldehyde for 24 h at 4°C, embedded in paraffin and sectioned at 5 μm for terminal deoxynucleotidyl transferase dUTP nick-end labeling (TUNEL) staining and Nissl staining.

### Real-Time Quantitative Polymerase Chain Reaction

miRNA-134 levels were measured by RT-qPCR with Taqman probes as previously described (Aronica et al., 2010; Guo M. et al., 2014). Total RNA was extracted from the cells by using TRIzol (Invitrogen) according to the manufacturer’s instructions, and cDNA was synthesized using a TaqMan miRNA reverse-transcription kit (Applied Biosystems, Foster City, CA, United States) following the manufacturer’s instruction. PCR system (10 μL) contained 5 μL of Taq PCR Master Mix, 1 μL of template cDNA, and 0.2 μL of forward and reverse primers of the target gene, miRNA-134 and reference gene U6. RT-qPCR was carried out on ABI 7500 system (Applied Biosystems, Foster City, CA, United States). The cycle threshold (Ct) values were calculated using the SDS 1.4 software (Applied Biosystems, Foster City, CA, United States), and the miR-134 expression was normalized with U6 SnRNA using the 2^−Δ Δ Ct^ method.

### Western Analysis

Protein isolation from hippocampal tissues was performed as previously (Zhou et al., 2010) described for either total cell isolates or cytosolic subcellular fractionated samples. Protein fraction was collected and homogenized on ice in RIPA buffer with protein concentration assessed and normalized with a Bradford protein assay kit (Beyotime, Shanghai, China). The following primary antibodies were used: anti-CHOP (1:200, Abcam), anti-CREB (1:200, Abcam), anti-Bim (1:200, Abcam), and anti-Cytochrome C (1:300, Abcam), anti-cleaved-caspase-3 (1:500, Cell Signaling Technology). Primary antibody detection was performed using secondary antibodies conjugated to horseradish peroxidase (Jackson Immuno Research). GAPDH expression in the same membrane was simultaneously determined as an internal reference. Western blots were imaged with a luminescent analyzer (GE Healthcare Bio-Sciences AB, Uppsala, Sweden) and quantification performed using Image Quant TL (GE Healthcare Bio-Sciences AB, Uppsala, Sweden).

### TUNEL Staining

After 24 h of lorazepam administration, TUNEL staining was performed on hippocampal sections to assess neuronal death (*n* = 5/group). TUNEL staining was performed using an *in situ* Cell Death Detection Kit (Roche), according to the manufacturer’s protocol (Wang et al., 2011). The sections were post-fixed in ethanol-acetic acid (2:1 vol/vol) and rinsed. Afterward, the sections were incubated with proteinase K (100 μg/mL), rinsed, incubated in 3% hydrogen peroxide, permeabilized with 0.5% Triton X-100, rinsed again, and incubated in the TUNEL reaction mixture. The sections were rinsed and visualized using Converter-POD with 0.03% DAB. Mayer’s hematoxylin (Dako, Glostrup, Denmark) was used as a counterstain, and sections were mounted onto gelatin-coated slides. The slides were air-dried overnight at room temperature, and coverslipped using Permount (Fisher Scientific). The numbers of TUNEL-positive cells in the hippocampal CA3 region were counted under a light microscope with 400 × magnification (Olympus, Tokyo, Japan). The extent of brain damage was evaluated by the average number of TUNEL-positive cells counted in 10 microscopic fields per group with square sampling regions (200 μm × 130 μm) and they were expressed as the number of cells per mm^2^ in the hippocampal CA3 region.

### Nissl Staining

Hippocampal neurons were visualized by Nissl staining (Zhang et al., 2002). Rats were euthanized with isoflurane 2 months after induction of SE, followed by sectioning and mounting of hippocampal slices. Sections were rehydrated in distilled water and stained with 0.5% crystal violet solution for 10 min. The brain slices in each section were imaged by the bright field microscopy (400×, Leica DM5000B). Normal neurons with visible nuclei in the CA3 subfield of hippocampal area were manually circled and counted within a unbiased counting fields (200 μm × 130 μm) per group. And they were expressed as the number of cells per mm^2^ in the hippocampal CA3 region. Neuronal numbers within the grid were evaluated from five brain sections per rat at different section depths for both the right and left sides of the brain.

### TIMM Staining

TIMM staining was performed as previously described (Xie et al., 2011). Serial coronal sections (8 μm) were collected with a cryocut (Leica VT 1000S). Every fifth section was collected on slides and dried at room temperature overnight. For the visualization of MFS, sections were developed using the following solution: 120 mL of 50% gum arabic, 10 mL of 47% sodium citrate buffer, 10 mL of 51% citrate buffer, 60 mL of 5.78% hydroquinone and 212.25 mg silver nitrate. The physical development was performed in the dark at room temperature for 120 min. The sections were rinsed in tap water, then dehydrated, cleared, and coverslipped in Entellan (107961.0100; Merck). Evaluation of these stains were conducted in accordance with the methodology outlined in Shetty and Turner (1997). The molecular layer, especially in naive animals, was divided in three parts of equal size, including the inner, middle and outer molecular layers (IML, MML, and OML, respectively). As the border between different layers is difficult to identify, the density of Timm-positive staining was made in the whole molecular layers by image J analysis software. Density measurements were evaluated from 10 brain sections per rat 400 mm^2^ apart for both the right and left sides of the brain.

### Electroencephalogram (EEG) and Analysis

For recoding of EEG, experimental mice were anesthetized with pentobarbital sodium (50 mg/kg) and placed in stereotaxic frame. Two skull-mounted recording electrodes were placed bilaterally on the duramater of the fronto-parietal cortex. All the electrodes were assembly fixed in place with dental cement during the surgical process. EEG setting: filter setting is the following. The high cut filter was 50 Hz and the low cut filter was 1.5 Hz. The time-constant was 0.1. Alternating current (a.c.) filters (50 Hz) were on. Continuous (24 h per day) EEG data were collected for 14 consecutive days after SE. EEG data were reviewed and manually scored by an observer unaware of experimental treatment with epileptic seizures defined as high frequency (>5 Hz) high amplitude (>2 × baseline) polyspike discharges of ≥ 5 s duration. The analysis of EEG frequency and amplitude was performed and analyzed by LabChart Pro v7 software (ADInstruments, Oxford, United Kingdom). The EEG results show the number of spikes per epoch (10 s) in SE and SE+ant-134 groups. EEG waveforms had amplitudes of 500 μV and frequencies ranging between 0 and 75 Hz.

### Analysis of Spontaneous Seizures by Continuous Video Monitoring

Animals distinguished by ear clips were housed in clear Perspex cages. Webcam-style cameras connected to laptop computers were placed 40 cm from cages in a room equipped with safe lights for night-time recordings. Videos from 5 days continuous (24 h per day) monitoring periods during weeks 3–4 post-SE were reviewed by an observer blind of experimental treatment. Behavioral analysis of spontaneous seizures was conducted by continuous video monitoring (*n* = 4/group). Only these rats graded 4 and above by Racine Scale, with typical seizure character such as continuous rearing and falling, severe tonic-clonic seizures, were recorded. As suggested, a new graph describing seizure frequency for all animals/groups was added. Graphs showed the average seizure attack times of individual animals on each day during the 5 days continuous video monitoring following SE with vehicle group and Ant-134 mice. Average seizure attack times of individual animals in each group were calculated and compared using the *t-*test.

### Statistical Analyses

Data analyses were performed using GraphPad InStat version 3.00 (GraphPad Software, San Diego, CA, United States). An unpaired, two-tailed Student’s *t*-test was used for comparisons between two groups. Multiple-group comparisons were performed using either one-way analysis of variance (ANOVA) where appropriated, followed by Turkey’s HSD *post hoc* multiple comparison test for two-way comparisons. All data are presented as the mean ± standard error of the mean (SEM). A value of *p* < 0.05 was considered statistically significant.

## Results

### *Status Epilepticus* Regulates miR-134 Expression

**FIGURE 1:**
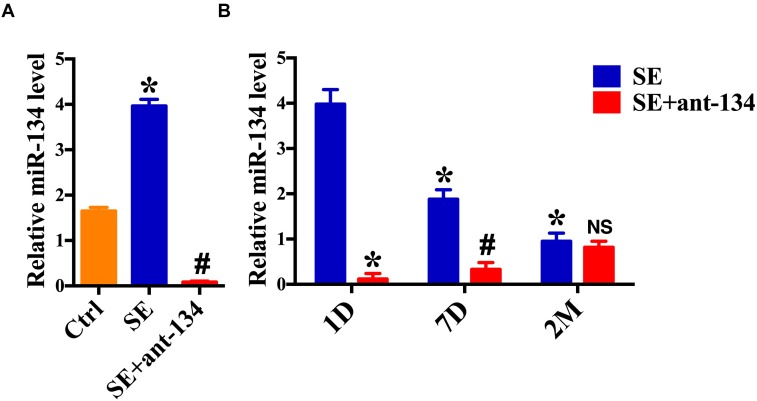
miR-134 is regulated by status epilepticus (SE). **(A)** RT-qPCR measurement of miR-134 levels in hippocampi from control rats (*n* = 6), SE+ant-134 rats (*n* = 6) and rats subjected to SE (*n* = 6) at 24 h after SE. ANOVA; ^∗^*p* < 0.05 compared with Ctrl, #*p* < 0.05 compared with SE. **(B)** RT-qPCR measurement of miR-134 levels in hippocampi from SE+ant-134 (*n* = 6) and SE rats (*n* = 6) at various time points. ANOVA; ^∗^*p* < 0.05 compared with SE after 1 day; #*p* < 0.05 compared with SE at 7 days. NS. No significant.

We first demonstrated that miR-134 levels were influenced by epileptic brain activity in rodents. Real-time quantitative PCR (RT-qPCR) revealed that 24 h following induction of SE there was an increase in miR-134 levels in the whole hippocampus, as compared to the control group. Furthermore, miR-134 levels were relatively decreased in the SE+ant-134 group (Figure 1A). 24 h after 0.12 nmol of miR-134 antagonist was injected into the rat ventricle, there was a significant decrease of miR-134. Recovery of miR-134 expression levels began after 7 days, consistent with other tissues (Krutzfeldt et al., 2005), and fully recovered after 2 months (Figure 1B).

### miR-134 Antaogmirs Resultant Neuronal Death

**FIGURE 2:**
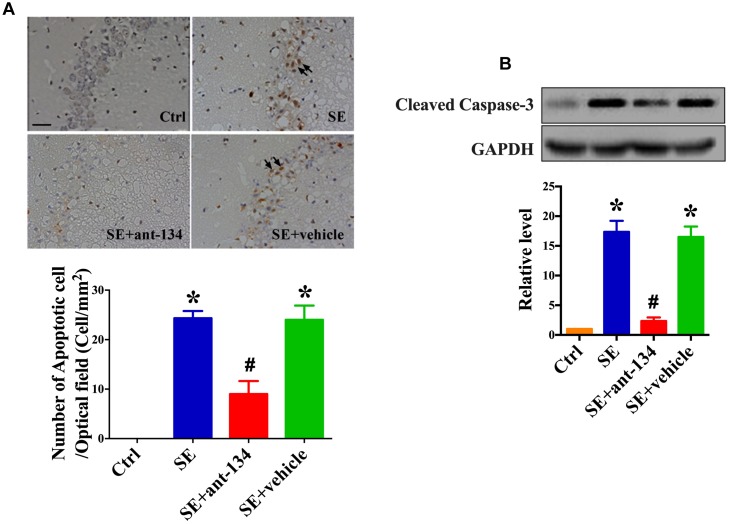
miR-134 antagomirs reduce neuronal death. **(A)** The representative images of TUNEL stained hippocampal CA3 sections and quantification of TUNEL-positive cells in control (*n* = 5), SE (*n* = 5), SE+ant-134 (*n* = 5), and SE+vehicle (*n* = 5) groups at 24 h post-SE. The arrows indicated the apoptosis cells. Scale bar = 20 μm. **(B)** Western blot analysis of cleaved Caspase-3 and the quantification result. ANOVA; ^∗^*p* < 0.05 compared with control group, ^#^*p* < 0.05 compared with SE+vehicle group.

We next assessed neuronal death in hippocampal sections from SE, SE+ant-134, and SE+vehicle group rats. SE and SE+vehicle group rats displayed extensive lesions and TUNEL-positive staining in the CA3 subfield of the hippocampus (Figure 2A). However, ant-134 administration dramatically reduced the number of seizure-induced TUNEL-positive neurons (Figure 2A). Consistently, cleaved Caspase-3, the hallmark of apoptosis was downregulated after silencing miR-134 compared with the SE models (Figure 2B).

### Silencing miR-134 Restores CREB Expression and Inhibits ER Stress-Mediated Neuronal Death

**FIGURE 3:**
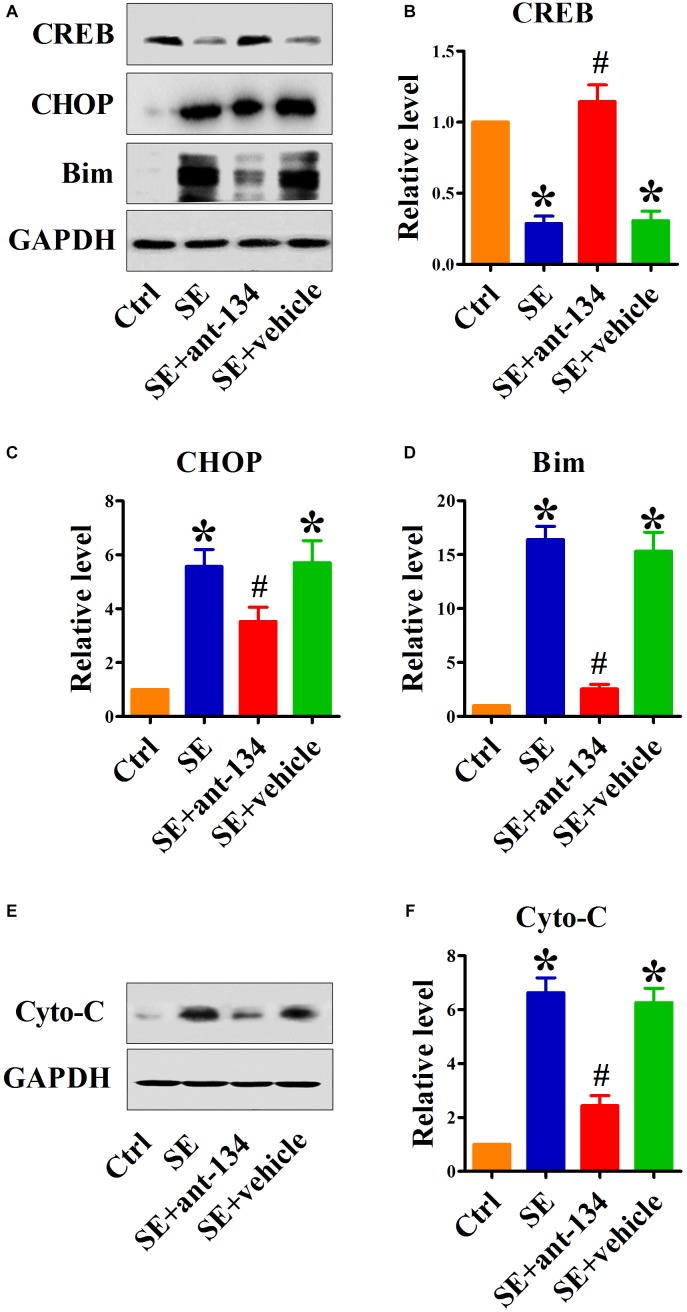
Silencing miR-134 restores CREB expression and inhibits ER stress-mediated neuronal apoptosis. **(A)** Representative Western blot showing CREB, CHOP and Bim protein levels in whole cells of control (*n* = 6), SE (*n* = 6), SE+ant-134 (*n* = 6), and SE+vehicle (*n* = 6) rats at 24 h after status epilepticus (SE). Quantification of **(B)** CREB, **(C)** CHOP, and **(D)** Bim protein levels. Cytochrome C **(E)** protein levels in cytoplasm and **(F)** quantification analysis. GAPDH was included as a loading control. ANOVA; ^∗^*p* < 0.05 compared with control; #*p* < 0.05 compared with SE+vehicle.

To determine the function of miR-134 in ER stress-induced neuronal apoptosis, we investigated the effect of miR-134 antagonists on the expression of relevant proteins. It has been shown that miR-134 serves as a regulator of CREB expression. We found that CREB protein levels were downregulated in the hippocampus after KA-induced SE. Analysis of the hippocampus in SE+ant-134 group rats showed upregulation of CREB protein levels compared with SE+vehicle group rats (Figure 3A,B).

We next specifically interrogated the role of ant-134 on SE-mediated ER stress. As expected, SE dramatically increased expression of the ER stress marker CHOP. Compared with the SE group, the SE+ant-134 group had decreased CHOP protein levels, while CHOP expression in the SE+vehicle group did not change (Figure 3C).

Because CHOP is a known transcriptional inducer of Bim, to investigate the influence of silencing miR-134 on ER stress-induced apoptosis, we quantified the pro-apoptotic proteins Bim and cytoplasmic cytochrome C (cyto-C). Ant-134 inhibited the increase of Bim (Figure 3D) and cyto-C protein levels (Figure 3E,F) induced by SE, while the vehicle injection did not block the increase in Bim and cyto-C protein levels.

### miR-134 Antagomir Administration Rescues KA-Induced Hippocampal Injury

**FIGURE 4:**
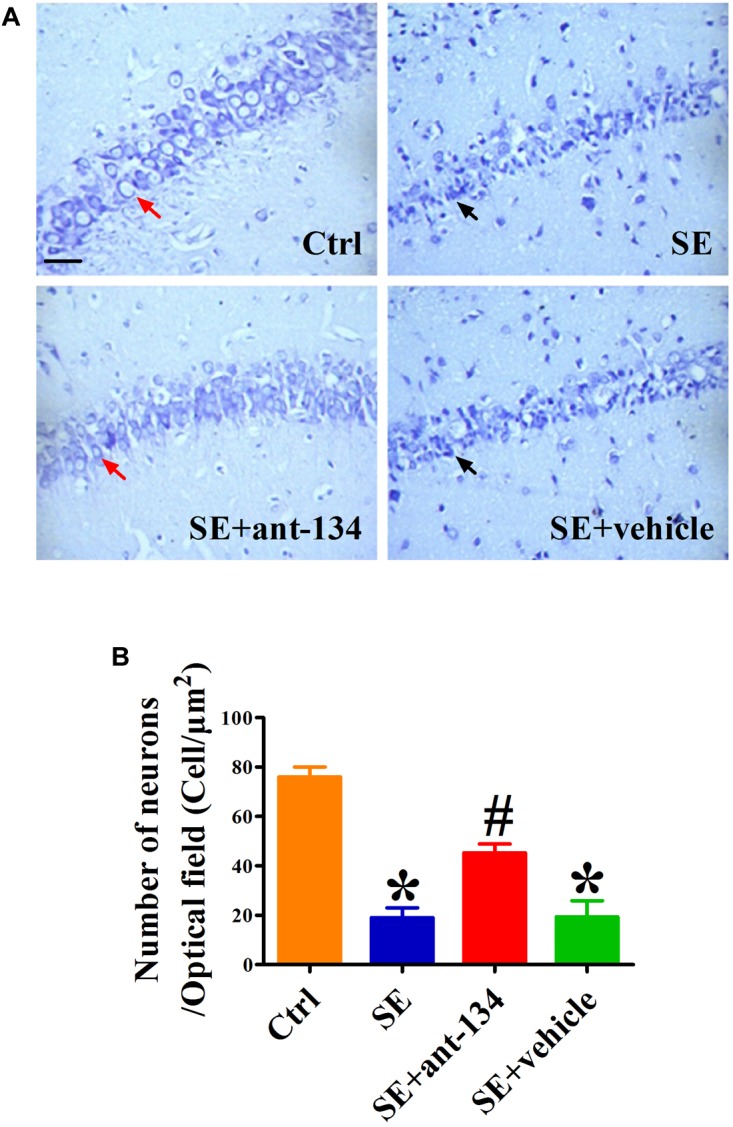
Hippocampal cytoarchitecture visualized with Nissl staining. **(A)** The representative images of Nissl stained CA3 sections of rat brains from control (*n* = 4), SE (*n* = 4), SE+vehicle (*n* = 4), and SE+ant-134 (*n* = 4) groups at 2 months following SE. **(B)** Quantification of number of neurons in CA3. The red arrows indicated the normal neurons. The apoptosis cells were indicated by the black arrows. Scale bar = 20 μm. ANOVA; ^∗^*p* < 0.05 compared with control group, ^#^*p* < 0.05 compared with SE+vehicle group.

We used Nissl staining to evaluate hippocampal architecture in each of our experimental groups at 2 months post KA administration. After counting the number of neurons in CA3 and comparing these values across groups, we found that the KA-induced lesion caused significant CA3 neuronal degeneration (Figure 4A). However, this degeneration could be rescued by administration of ant-134, as the total number of neurons present in the SE+ant-134 group was increased relative to that of the SE+vehicle group (Figure 4B). These data suggest that concomitant silencing of miR-134 during seizure induction can persistently attenuate neuronal death in CA3.

### miR-134 Antagomir Treatment Reduces Seizure-Induced Mossy Fiber Sprouting (MFS)

**FIGURE 5:**
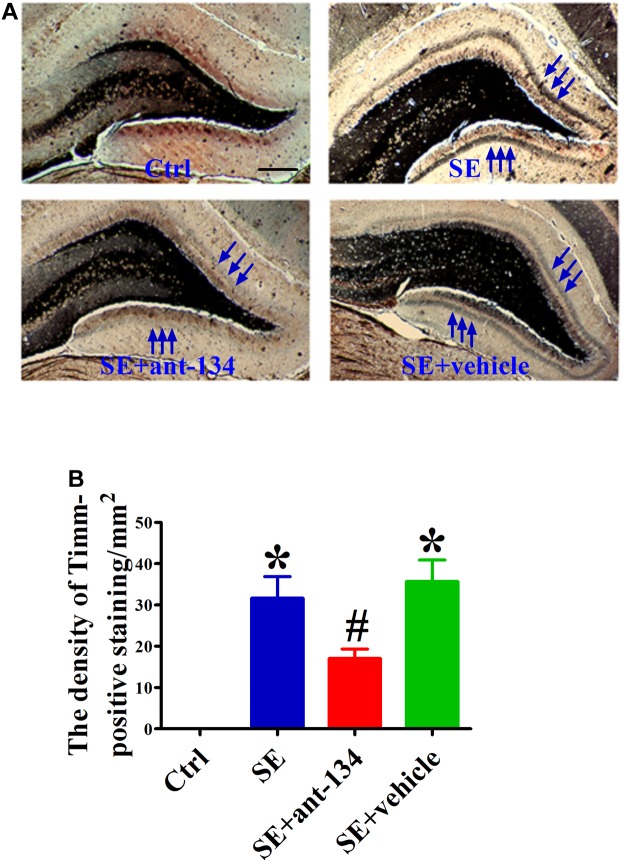
Comparison of KA-lesion induced MFS using Timm’s staining in DH. **(A)** The representative photomicrographs of Timm’s staining in the hippocampus of rat brain sections from control (*n* = 4), SE (*n* = 4), SE+ant-134 (*n* = 4), and SE+vehicle (*n* = 4) groups at 24 h after SE. Scale bars = 200 μm. **(B)** Quantitative data for density of sprouting into the molecular layer. Scale bars = 200 μm. ANOVA; ^∗^*p* < 0.05 compared with SE group; #*p* < 0.05 compared with SE+vehicle.

In both human and animal epilepsy models, DG granule cell mossy fibers undergo significant reorganization of their terminal projections. We used Timm’s staining, which specifically labels mossy fiber synaptic boutons by virtue of their high zinc content, to identify aberrant mossy fiber sprouting (MFS) following KA-mediated SE induction. We also assessed the effect of ant-134 infusion on KA-induced MFS at 2 months post-lesion. The SE and SE+vehicle groups exhibited significant MFS that mainly projected into the inner molecular (Figure 5A). As expected, the band of Timm staining was visibly reduced in the SE+ant-134 group (Figure 5B).

### miR-134 Antagomirs Protect the Later Occurrence of Spontaneous Seizures

**FIGURE 6:**
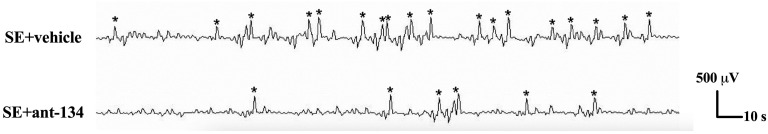
Representative EEG recordings of abnormal spikes from SE (*n* = 4) and SE+ant-134 (*n* = 4) groups at 2 months. Asterisk indicates the abnormal spikes of epileptic brain. The duration of the recording time is 10 s and the amplitude scale is 500 μV.

Hippocampal activity was monitored with electroencephalography (EEG) at 2 months after KA-lesion. The EEG results showed that the number of spikes per epoch (10 s) in SE+ant-134 groups was dramatically decreased compared with the SE+vehicle group (Figure 6). Additionally, the SE+ant-134 group presented with a significantly lower frequency (24.8 ± 7.2/min) of abnormal spikes than that of the SE group (57.8 ± 6.4/min). These results suggest that miR-134 antagomirs may reduce abnormal neuronal discharge in the epileptic hippocampus over the long-term.

### The Antagomirs of miR-134 Relieved the Seizure Behavior of Rats

**FIGURE 7:**
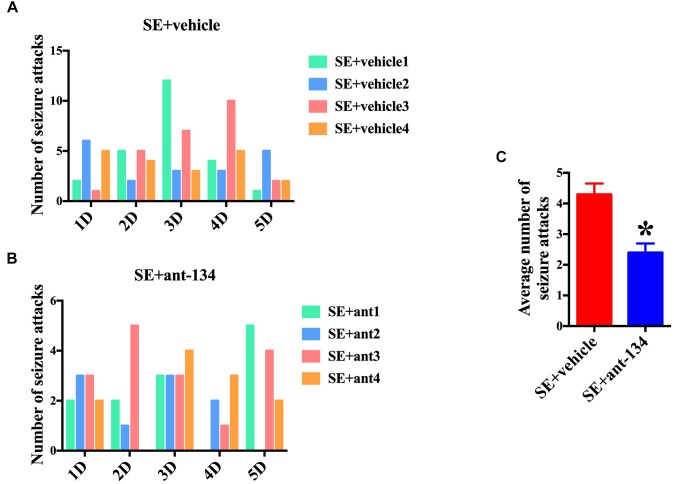
Behavioral analysis of rats between miR-134 antagomirs and vehicle group. Graphs show the number of seizure behaviors each day for individual animals during 5 days continuous video monitoring following SE (*n* = 4) for **(A)** vehicle (*n* = 4) and **(B)** Ant-134 (*n* = 4) group. **(C)** The average number of seizure behaviors per rat during the monitoring periods. *t*-Test; ^∗^*p* < 0.05 compared with SE+vehicle.

We also scored seizure the activity of rats at 2 months post KA-induced SE. Four rats were selected in each group and uninterrupted video was monitored. The number of seizure attacks (Racine Score 1∼5) of each rat in SE+vehicle group (Figure 7A) and SE+ant-134 group (Figure 7B) was scored during the 5 days. The total number of seizure attacks of SE+ant-134 group (48 times) was significantly decreased than the SE group (92 times) and SE+vehicle group (86 times). The average seizure level of SE+ant-134 group was downregulated than SE+vehicle group (Figure 7C). These results showed that ant-134 attenuated the seizure score, which can serve as a potential treatment for epilepsy.

## Discussion

This study demonstrates that inhibition of miR-134 effectively protects hippocampal neurons from status epilepticus-induced neuronal death in an animal model of epilepsy. MiR-134 inhibition also attenuates aberrant MFS from granule cells in the dentate gyrus (DG), which may in the long-term lead to decreased diseased neuronal discharges in the epileptic brain. These findings suggest that silencing miR-134 may be an appropriate intervention to treat status epilepticus (SE), attenuate seizure-related structural damage to the hippocampus, and prevent the induction of later spontaneous seizure events. Additionally, we find that miR-134 inhibition restores CREB expression and decreases CHOP and Bim expression, genes that are relevant to ER stress-mediated apoptosis. Therefore, the likely mechanism whereby inhibition of miR-134 affects the epileptic brain is through reducing CREB expression. This study not only supports the prospect of exploring miR-134 antagomirs clinically, but also provides a basis for discovering a detailed mechanism of action.

Epilepsy is a common neurological disorder that has an estimated prevalence of 50 million people and accounts for around 1% of the global disease burden. While epilepsy is often spontaneous, it is possible that up to 50% of cases are acquired, and this number increases as people age. Like cerebrovascular diseases and neurodegeneration, epileptic brain tissue exhibits both increased ROS production and chronic inflammation, and it is likely that this inflammatory state contributes to, or even initiates, the disorder (Glass et al., 2010). Although the full mechanism remains to be elucidated, there is some evidence that mitochondrial complexes I and II as well as membrane-bound NADPH oxidases play a role in elevating ROS levels (Kumagai and Sumi, 2007). Furthermore, ROS-mediated oxidative stress likely plays an important role in epilepsy symptoms get worse and neuronal death. We have previously shown that that ant-134 treatment reduces hippocampal oxidative stress by assaying SOD activity and quantifying MDA and 4_HNE levels (Sun et al., 2017). MicroRNAs (miRNAs) play important roles in the pathogenesis of neurologic disorders and it is therefore unsurprising they have demonstrated promise as therapeutics. For instance, in a pilocarpine model of SE, miR-134 knockdown was shown to effectively suppress seizures and facilitate neuroprotection (Jimenez-Mateos et al., 2015). Increased miR-134 expression reduces dendritic spine volume of hippocampal neurons, and this anatomical change has been identified as a consistent marker of epilepsy. Apart from the apparent benefits of using a miR-134 antagonist (ant-134) to reduce seizure frequency and intensity, in this study we wanted to determine whether ant-134 could reduce neuronal injury in a SE model in rats. Previous studies reported that when produced via intra-amygdala microinjection of KA, SE develops following the propagation of seizures through the entorhinal cortex and perforant pathway to the hippocampus. In the present study, we observed that the number of apoptotic cells in hippocampal CA3 as assed by TUNEL staining was significantly reduced by ant-134 administration as compared with controls. This difference also appeared in the western blot analyses of proapoptotic proteins Bim and cytochrome C. From these results, we speculate that miR-134 inhibition can play a protective role in epilepsy-induced damage of hippocampal neurons.

To determine the mechanism by which miR-134 inhibition reduces neuronal damage, we decided to investigate the role of the ER, which can engender a stress response that causes apoptosis and contributes to pathologies, including epilepsy. The ER plays critical roles in calcium storage as well as protein folding, assembly, and modification. ER stress can be induced by an array of pathophysiological conditions, including ischemia, oxidative stress, or infection (Ron, 2002). If ER stress is overwhelming, pro-apoptotic signals dominate, and cells undergo apoptosis. In particular, ER stress is partially mediated by an increased expression of the pro-apoptotic transcription factor C/EBP homologous protein (CHOP), which is thought to play a critical role in apoptosis in a variety of cardiac and neurological disorders (Fu et al., 2010; Zou et al., 2012). CHOP, in turn, stimulates the cell-death inducer Bim. Thus, apoptosis following ER stress may result from increased transcriptional efficacy of CHOP, which in turn upregulates expression of the pro-apoptotic Bim protein (Puthalakath et al., 2007). This mechanism is consistent with our results. Overall, we found that downregulating miR-134 does indeed reduce hippocampal CHOP and Bim expression. However, it is unlikely that reducing Bim activity is the only mechanism by which ant-134 reduces neuronal damage, as many other pro-apoptotic proteins and pathways are activated following seizures. Furthermore, a research showed after seizures, hippocampal CA3 neurodegeneration was reduced in Bim-deficient mice, while neocortex damage in Bim-deficient mice was comparable with that in wild-type mice. These results suggested the region-specific differential contributions of Bim to seizure-induced neuronal death (Murphy et al., 2010). To further understand the mechanism of ER stress-induced neuron damage, we turned our attention to CREB, a leucine zipper family transcription factor that is ubiquitously expressed (Yamashima, 2012). CREB controls proliferation and differentiation during neural development, and also functions as a key regulator of neuronal plasticity, learning, and memory in the adult brain. CREB is neuroprotective against various insults, including ischemia, traumatic injury, and neurodegeneration (Shaywitz and Greenberg, 1999; Zanassi et al., 2001; Zhu et al., 2004). In fact, there are several studies that implicate CREB activation in adult neurogenesis, including in the dentate gyrus following ischemic injury. Furthermore, Qiu et al. (2013) have shown that CREB knockdown reduced the protective ability of cocaine- and amphetamine-regulated transcript (CART) protein to inhibit cerebral ischemia/reperfusion-induced ER stress and neuronal apoptosis. Additionally, Li et al. (2015) have shown that nicotinamide protects hepatocytes against ER stress via activation of the cAMP/PKA/CREB pathway. Regarding the relationship between CREB and miR-134, previous studies indicate that downregulation of miR-134 increases CREB expression, which attenuates H_2_O_2_-induced RGC apoptosis (Shao et al., 2015). Also, resveratrol might function as a neuroprotective agent by reducing miR-134 and increasing CREB expression (Zhao et al., 2013). In this study, we demonstrate that miR-134 inhibition results in CREB Increased expression and a decrease in ER stress-induced apoptosis. Our results substantiate the idea that CREB plays a crucial role in preventing hippocampal neuronal injury induced by epilepsy. Given these findings, as well as CREB’s importance throughout the nervous system, CREB may be an ideal target for future therapeutic intervention in epilepsy, among other neurological disorders.

Studies conducted with human patients and animal models have suggested that mossy fibers that lose their normal synaptic targets following degeneration of CA3 pyramidal neurons and DG hilus interneurons may begin to sprout abnormally (Mathern et al., 1996; Shetty and Turner, 1997; Rao et al., 2006). Given the importance of neuronal degeneration in producing MFS, one of the main characteristic anatomical phenotypes of epilepsy, it was worth investigating whether the decreases in neuronal death in injured hippocampi following miR-134 antagomir treatment can contribute to reducing MFS. Indeed, our results indicate that anti-134 treatment reduced aberrant MFS throughout the dentate gyrus. Previous research has shown that miR-134 antagomirs suppress seizures by modulating dendritic spine density (Jimenez-Mateos et al., 2012) and size (Schratt et al., 2006). However, most studies have focused mainly on the transient effects of anti-miR-134 treatment, with follow-up ranging from 3 to 14 days post SE induction. Our results not only further support the antiepileptogenic effect of miR-134 antagomirs, but also indicate that this protective effect can last up to 2 months after KA-induced SE. This long-term effect suggests that miR-134 antagomirs may have therapeutic potential in the treatment of epilepsy.

Overall, the present study establishes a significant role for miR-134 antagomirs in the alleviation of hippocampal neuron injury following KA-induced SE, which has been shown to contribute to the onset and occurrence of spontaneous seizures and other neurological disorders. However, future work should investigate the impact of miR-134 on characteristic epilepsy phenotypes such as aberrant neuronal circuitry, which gives rise to epilepsy symptoms like impaired spatial navigation and memory. Additionally, it will be crucial to determine the clinical feasibility of incorporating miR-134 into an epilepsy treatment regimen, taking into account limitations such as the blood-brain barrier.

## Ethics Statement

This study was carried out in accordance with the recommendations of ‘National Institutes of Health guide for the care and use of laboratory animals.’ The protocol was approved by the ‘Research Ethics Committee of Harbin Medical University.’

## Author Contributions

XGa and MG wrote the manuscript and analyzed the data. DM and LG performed the animal experiments. FS made great contribution to the revision of the manuscript. YC and YZ performed the RT-qPCR experiments. XW and XGu conducted Western Blot experiments. JS and SQ designed the study and revised the manuscript.

## Conflict of Interest Statement

The authors declare that the research was conducted in the absence of any commercial or financial relationships that could be construed as a potential conflict of interest.

## References

[B1] Aronica E. Fluiter K. Iyer A. Zurolo E. Vreijling J. van Vliet E. A. (2010). Expression pattern of miR-146a, an inflammation-associated microRNA, in experimental and human temporal lobe epilepsy. *Eur. J. Neurosci.* 31 1100–1107. 10.1111/j.1460-9568.2010.07122.x 20214679

[B2] Ben-Ari Y. (1985). Limbic seizure and brain damage produced by kainic acid: mechanisms and relevance to human temporal lobe epilepsy. *Neuroscience* 14 375–403. 10.1016/0306-4522(85)90299-4 2859548

[B3] Cai Z. Li S. Li S. Song F. Zhang Z. Qi G. (2016). Antagonist targeting microRNA-155 protects against lithium-pilocarpine-induced status epilepticus in c57bl/6 mice by activating brain-derived neurotrophic factor. *Front. Pharmacol.* 7:129. 10.3389/fphar.2016.00129 27303295 PMC4885878

[B4] Fu H. Y. Okada K. Liao Y. Tsukamoto O. Isomura T. Asai M. (2010). Ablation of C/EBP homologous protein attenuates endoplasmic reticulum-mediated apoptosis and cardiac dysfunction induced by pressure overload. *Circulation* 122 361–369. 10.1161/CIRCULATIONAHA.109.917914 20625112

[B5] Glass C. K. Saijo K. Winner B. Marchetto M. C. Gage F. H. (2010). Mechanisms underlying inflammation in neurodegeneration. *Cell* 140 918–934. 10.1016/j.cell.2010.02.016 20303880 PMC2873093

[B6] Guo J. Wang H. Wang Q. Chen Y. Chen S. (2014). Expression of p-CREB and activity-dependent miR-132 in temporal lobe epilepsy. *Int. J. Clin. Exp. Med.* 7 1297–1306. 24995086 PMC4073747

[B7] Guo M. Jiang Z. Zhang X. Lu D. Ha A. D. Sun J. (2014). miR-656 inhibits glioma tumorigenesis through repression of BMPR1A. *Carcinogenesis* 35 1698–1706. 10.1093/carcin/bgu030 24480809

[B8] Henshall D. C. Simon R. P. (2005). Epilepsy and apoptosis pathways. *J. Cereb. Blood Flow Metab.* 25 1557–1572. 10.1038/sj.jcbfm.9600149 15889042

[B9] Huang W. Liu X. Cao J. Meng F. Li M. Chen B. (2015). mir-134 regulates ischemia/reperfusion injury-induced neuronal cell death by regulating CREB signaling. *J. Mol. Neurosci.* 55 821–829. 10.1007/s12031-014-0434-0 25316150

[B10] Huang Y. Guo J. Wang Q. Chen Y. (2014). MicroRNA-132 silencing decreases the spontaneous recurrent seizures. *Int. J. Clin. Exp. Med.* 7 1639–1649. 25126160 PMC4132124

[B11] Jimenez-Mateos E. M. Engel T. Merino-Serrais P. Fernaud-Espinosa I. Rodriguez-Alvarez N. Reynolds J. (2015). Antagomirs targeting microRNA-134 increase hippocampal pyramidal neuron spine volume in vivo and protect against pilocarpine-induced status epilepticus. *Brain Struct. Funct.* 220 2387–2399. 10.1007/s00429-014-0798-5 24874920

[B12] Jimenez-Mateos E. M. Engel T. Merino-Serrais P. McKiernan R. C. Tanaka K. Mouri G. (2012). Silencing microRNA-134 produces neuroprotective and prolonged seizure-suppressive effects. *Nat. Med.* 18 1087–1094. 10.1038/nm.2834 22683779 PMC3438344

[B13] Krutzfeldt J. Rajewsky N. Braich R. Rajeev K. G. Tuschl T. Manoharan M. (2005). Silencing of microRNAs in vivo with ’antagomirs’. *Nature* 438 685–689. 10.1038/nature04303 16258535

[B14] Kumagai Y. Sumi D. (2007). Arsenic: signal transduction, transcription factor, and biotransformation involved in cellular response and toxicity. *Annu. Rev. Pharmacol. Toxicol.* 47 243–262. 10.1146/annurev.pharmtox.47.120505.105144 17002598

[B15] Li J. Dou X. Li S. Zhang X. Zeng Y. Song Z. (2015). Nicotinamide ameliorates palmitate-induced ER stress in hepatocytes via cAMP/PKA/CREB pathway-dependent sirt1 upregulation. *Biochim. Biophys. Acta* 1853 2929–2936. 10.1016/j.bbamcr.2015.09.003 26352206 PMC5445659

[B16] Mathern G. W. Babb T. L. Leite J. P. Pretorius K. Yeoman K. M. Kuhlman P. A. (1996). The pathogenic and progressive features of chronic human hippocampal epilepsy. *Epilepsy Res.* 26 151–161. 10.1016/S0920-1211(96)00052-6 8985697

[B17] Murphy B. M. Engel T. Paucard A. Hatazaki S. Mouri G. Tanaka K. (2010). Contrasting patterns of bim induction and neuroprotection in bim-deficient mice between hippocampus and neocortex after status epilepticus. *Cell Death Differ.* 17 459–468. 10.1038/cdd.2009.134 19779495 PMC2950266

[B18] Olney J. W. Collins R. C. Sloviter R. S. (1986). Excitotoxic mechanisms of epileptic brain damage. *Adv. Neurol.* 44 857–877. 3706027

[B19] Ouyang Y. B. Xu L. Lu Y. Sun X. Yue S. Xiong X. X. (2013). Astrocyte-enriched miR-29a targets PUMA and reduces neuronal vulnerability to forebrain ischemia. *Glia* 61 1784–1794. 10.1002/glia.22556 24038396 PMC3810393

[B20] Puthalakath H. O’Reilly L. A. Gunn P. Lee L. Kelly P. N. Huntington N. D. (2007). ER stress triggers apoptosis by activating BH3-only protein Bim. *Cell* 129 1337–1349. 10.1016/j.cell.2007.04.027 17604722

[B21] Qiu B. Hu S. Liu L. Chen M. Wang L. Zeng X. (2013). CART attenuates endoplasmic reticulum stress response induced by cerebral ischemia and reperfusion through upregulating BDNF synthesis and secretion. *Biochem. Biophys. Res. Commun.* 436 655–659. 10.1016/j.bbrc.2013.05.142 23770418

[B22] Racine R. J. Gartner J. G. Burnham W. M. (1972). Epileptiform activity and neural plasticity in limbic structures. *Brain Res.* 47 262–268. 10.1016/0006-8993(72)90268-5 4641271

[B23] Rao M. S. Hattiangady B. Reddy D. S. Shetty A. K. (2006). Hippocampal neurodegeneration, spontaneous seizures, and mossy fiber sprouting in the F344 rat model of temporal lobe epilepsy. *J. Neurosci. Res.* 83 1088–1105. 10.1002/jnr.20802 16493685

[B24] Ron D. (2002). Translational control in the endoplasmic reticulum stress response. *J. Clin. Invest.* 110 1383–1388. 10.1172/JCI16784 12438433 PMC151821

[B25] Schratt G. M. Tuebing F. Nigh E. A. Kane C. G. Sabatini M. E. Kiebler M. (2006). A brain-specific microRNA regulates dendritic spine development. *Nature* 439 283–289. 10.1038/nature04367 16421561

[B26] Shao Y. Yu Y. Zhou Q. Li C. Yang L. Pei C. G. (2015). Inhibition of miR-134 protects against hydrogen peroxide-induced apoptosis in retinal ganglion cells. *J. Mol. Neurosci.* 56 461–471. 10.1007/s12031-015-0522-9 25744098

[B27] Shaywitz A. J. Greenberg M. E. (1999). CREB: a stimulus-induced transcription factor activated by a diverse array of extracellular signals. *Annu. Rev. Biochem.* 68 821–861. 10.1146/annurev.biochem.68.1.821 10872467

[B28] Shetty A. K. Turner D. A. (1997). Fetal hippocampal cells grafted to kainate-lesioned CA3 region of adult hippocampus suppress aberrant supragranular sprouting of host mossy fibers. *Exp. Neurol.* 143 231–245. 10.1006/exnr.1996.6363 9056386

[B29] Sun J. Gao X. Meng D. Xu Y. Wang X. Gu X. (2017). Antagomirs targeting mirorna-134 attenuates epilepsy in rats through regulation of oxidative stress, mitochondrial functions and autophagy. *Front. Pharmacol.* 8:524. 10.3389/fphar.2017.00524 28848439 PMC5550691

[B30] Uttara B. Singh A. V. Zamboni P. Mahajan R. T. (2009). Oxidative stress and neurodegenerative diseases: a review of upstream and downstream antioxidant therapeutic options. *Curr. Neuropharmacol.* 7 65–74. 10.2174/157015909787602823 19721819 PMC2724665

[B31] Wang Q. Zhang X. Ding Q. Hu B. Xie Y. Li X. (2011). Limb remote postconditioning alleviates cerebral reperfusion injury through reactive oxygen species-mediated inhibition of delta protein kinase C in rats. *Anesth. Analg.* 113 1180–1187. 10.1213/ANE.0b013e31822b885f 21865497

[B32] Xie C. Sun J. Qiao W. Lu D. Wei L. Na M. (2011). Administration of simvastatin after kainic acid-induced status epilepticus restrains chronic temporal lobe epilepsy. *PLoS One* 6:e24966. 10.1371/journal.pone.0024966 21949812 PMC3176286

[B33] Yamashima T. (2012). ’PUFA-GPR40-CREB signaling’ hypothesis for the adult primate neurogenesis. *Prog. Lipid Res.* 51 221–231. 10.1016/j.plipres.2012.02.001 22390974

[B34] Zanassi P. Paolillo M. Feliciello A. Avvedimento E. V. Gallo V. Schinelli S. (2001). cAMP-dependent protein kinase induces cAMP-response element-binding protein phosphorylation via an intracellular calcium release/ERK-dependent pathway in striatal neurons. *J. Biol. Chem.* 276 11487–11495. 10.1074/jbc.M007631200 11139572

[B35] Zhang X. Cui S. S. Wallace A. E. Hannesson D. K. Schmued L. C. Saucier D. M. (2002). Relations between brain pathology and temporal lobe epilepsy. *J. Neurosci.* 22 6052–6061. 10.1523/JNEUROSCI.22-14-06052.2002 12122066 PMC6757939

[B36] Zhao Y. N. Li W. F. Li F. Zhang Z. Dai Y. D. Xu A. L. (2013). Resveratrol improves learning and memory in normally aged mice through microRNA-CREB pathway. *Biochem. Biophys. Res. Commun.* 435 597–602. 10.1016/j.bbrc.2013.05.025 23685142

[B37] Zhou Y. Lekic T. Fathali N. Ostrowski R. P. Martin R. D. Tang J. (2010). Isoflurane posttreatment reduces neonatal hypoxic-ischemic brain injury in rats by the sphingosine-1-phosphate/phosphatidylinositol-3-kinase/Akt pathway. *Stroke* 41 1521–1527. 10.1161/STROKEAHA.110.583757 20508187 PMC2917259

[B38] Zhu D. Y. Lau L. Liu S. H. Wei J. S. Lu Y. M. (2004). Activation of cAMP-response-element-binding protein (CREB) after focal cerebral ischemia stimulates neurogenesis in the adult dentate gyrus. *Proc. Natl. Acad. Sci. U.S.A.* 101 9453–9457. 10.1073/pnas.0401063101 15197280 PMC438997

[B39] Zou X. J. Yang L. Yao S. L. (2012). Endoplasmic reticulum stress and C/EBP homologous protein-induced bax translocation are involved in angiotensin II-induced apoptosis in cultured neonatal rat cardiomyocytes. *Exp. Biol. Med.* 237 1341–1349. 10.1258/ebm.2012.012041 23239445

